# Arrhythmia Classification of ECG Signals Using Hybrid Features

**DOI:** 10.1155/2018/1380348

**Published:** 2018-11-12

**Authors:** Syed Muhammad Anwar, Maheen Gul, Muhammad Majid, Majdi Alnowami

**Affiliations:** ^1^Department of Software Engineering, University of Engineering and Technology, Taxila, Pakistan; ^2^Department of Computer Engineering, University of Engineering and Technology, Taxila, Pakistan; ^3^Department of Nuclear Engineering, King Abdulaziz University, Jeddah, Saudi Arabia

## Abstract

Automatic detection and classification of life-threatening arrhythmia plays an important part in dealing with various cardiac conditions. In this paper, a novel method for classification of various types of arrhythmia using morphological and dynamic features is presented. Discrete wavelet transform (DWT) is applied on each heart beat to obtain the morphological features. It provides better time and frequency resolution of the electrocardiogram (ECG) signal, which helps in decoding important information of a quasiperiodic ECG using variable window sizes. RR interval information is used as a dynamic feature. The nonlinear dynamics of RR interval are captured using Teager energy operator, which improves the arrhythmia classification. Moreover, to remove redundancy, DWT subbands are subjected to dimensionality reduction using independent component analysis, and a total of twelve coefficients are selected as morphological features. These hybrid features are combined and fed to a neural network to classify arrhythmia. The proposed algorithm has been tested over MIT-BIH arrhythmia database using 13724 beats and MIT-BIH supraventricular arrhythmia database using 22151 beats. The proposed methodology resulted in an improved average accuracy of 99.75% and 99.84% for class- and subject-oriented scheme, respectively, using three-fold cross validation.

## 1. Introduction

Cardiac arrhythmias are a type of irregular heartbeats in which the heart rhythm is either too fast (tachycardia) or too slow (bradycardia). A small change in electrocardiogram (ECG) morphology or dynamics may lead to severe arrhythmia attacks, which can reduce the ability of the heart to pump blood and causes shorting of breath, pain in chest, tiredness, and loss of consciousness. There are several types of arrhythmia, and some of these are dangerous which may lead to cardiac arrest and sudden death if not detected and monitored in time [1]. There are other types of arrhythmias which are not essentially life-threatening, but still require proper analysis to avoid future clinical problems. A few categories of arrhythmia appear infrequently in the ECG signal and hence require long electrocardiogram recordings in order to be detected. A manual analysis of longer ECG recordings requires time and great effort. The automatic detection and classification of these arrhythmias offer great assistance to physicians [2, 3]. Moreover, early diagnosis of arrhythmias would help in proper treatment and support sustained life. Therefore, several techniques have been proposed for automatic detection and classification of various types of arrhythmia [4–10].

A method for classification of sixteen types of arrhythmia based on ECG morphology and dynamics was proposed in [11]. Morphological features were extracted using discrete wavelet transform (DWT) and independent component analysis (ICA), while ECG dynamic features were extracted by calculating RR interval. These features were then classified using support vector machine with an average accuracy of 99.6%. Five types of heartbeats, namely, normal, premature ventricular contractions (PVC), atrial premature contractions (APC), left bundle branch block (LBBB), and right bundle branch block (RBBB) were recognized in [4]. Stationary wavelet transform (SWT) was applied on each ECG signal to make it noise free. The high-order statistics of ECG signals and three timing intervals were considered as features, which were then fed to a hybrid bees algorithm for training the radial basis function and resulted in an average accuracy of 95.18%.

Some techniques rely on fiducial features, which are temporal and dynamic features that directly depend upon the ECG characteristics, e.g., wave onset point, peaks (maxima/minima), and offset [5]. Nonfiducial features that are directly derived from the fiducial features or obtained by segmenting the ECG signal into several parts have also been used [6]. These features are not found good enough when used independently for accurate classification of ECG arrhythmia. A combination of both fiducial and nonfiducial features was used for defining a biological marker for person identification [7, 12].

The extracted features have usually been analyzed either in time domain or frequency domain. Some of the most important time domain features, including RR interval, ST segment, and T height, require identification of key time points within the signal [13]. An approach for heartbeat classification with dynamic rejection thresholds was proposed using QRS morphology, frequency information, AC power of QRS detail coefficients, and RR intervals as features to represent ECG beats [8]. A support vector machine (SVM) was used to classify with an improved accuracy of 97.2% and minimum rejection cost. A combination of time and frequency information has also been used in [9, 11, 14–16], giving better extraction of information from a quasiperiodic ECG signal using wavelet transform, which provides a good time and frequency resolution. Five important types of arrhythmia, namely, nonectopic, ventricular ectopic, supraventricular ectopic, unclassifiable beats, and fusion betas, were analyzed and detected in [9]. Features were extracted using DWT, and only significant coefficients were selected by applying independent component analysis which in combination with neural network yielded an accuracy of 99.28%.

All methods discussed above have the following shortcomings:

1. Performances of most of the methods have been tested only on smaller data sets, and there is a need to verify their performance on larger databases
2. Selected classes of arrhythmia have been evaluated, and there is a need to test all arrhythmia classes
3. The classification accuracy on sparsely occurring arrhythmia classes is not good

In this paper, a novel technique for ECG beat classification of arrhythmia is proposed that considers a hybrid of enhanced morphological and dynamic features to overcome these shortcomings. Morphological features are obtained using DWT on each heartbeat. The resulting features consist of DWT approximation coefficient and detail coefficients at level 4. Independent component analysis is applied on both approximation and detail coefficients independently to extract only important coefficients. In addition, four types of RR interval features are calculated to represent the dynamic features of ECG heartbeats. Moreover, to enhance the dynamic features of ECG heartbeats corrupted with Gaussian noise, Teager energy operator (TEO) [17] is used. All these features are combined and fed to a neural network (NN) for automatic classification of heartbeats into different arrhythmia types according to both class-oriented scheme (18 classes) and subject-oriented scheme (5 classes).

The rest of the paper is organized as follows. Firstly, the proposed methodology is explained in detail in Section 2. Class- and subject-oriented evaluation of the proposed system is presented in Section 3, followed by conclusion in Section 4.

## 2. Methodology

**Figure 1:**
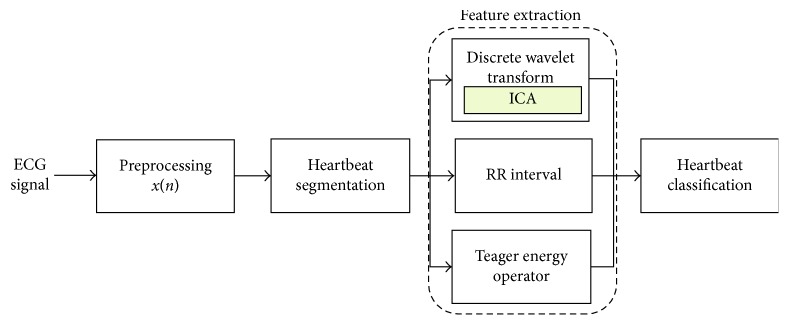
Block diagram of the proposed arrhythmia classification scheme using hybrid features.

The proposed methodology is presented in Figure 1, which consists of four major phases, namely, preprocessing, heartbeat segmentation, feature extraction, and feature classification. The details of these phases are presented in the following subsections.

### 2.1. ECG Signal Preprocessing

Mostly ECG signals are affected by baseline wander or power line interface (PLI). Different methods were introduced to remove these types of noises from the ECG signal [18]. Experiments showed that baseline wander significantly affects the detection of arrhythmia and makes the ECG signal analysis difficult for an expert [19]. PLI is a type of noise that occurs due to broken electrodes, offset voltages in the electrodes, movement of patient, respiration errors, or electrode resistance while recording the ECG signal. It is a low frequency noise and typically exists in the frequency range of 0–0.3 Hz. For baseline wander removal, the expected value of the signal was subtracted from the raw ECG signal to obtain the noise-free ECG signal using

(1) $$\begin{array}{c} x\left[n\right]=x_{\text{r}}\left[n\right]-\mu , \end{array}$$

where *x*[*n*] is the denoised signal, *x*_r_[*n*] represents the raw ECG signal, and *μ* is the expected value of the raw EEG signal.

### 2.2. Heartbeat Segmentation

**Figure 2:**
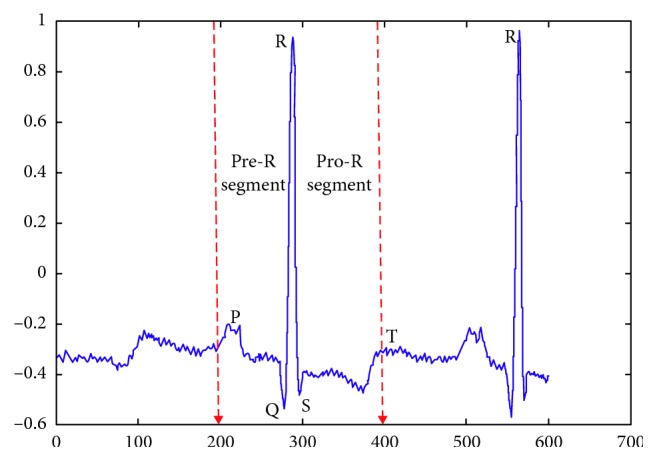
Heartbeat segmentation of ECG signal from MITDB database.

Three basic constituents of a heart cycle are QRS complex, T wave, and P wave, termed as fiducial points. The correct splitting of the ECG signal into heartbeat segments involves recognition of borders and peak locations of these fiducial points. The information about the R-peak locations given in the dataset was used to obtain these heartbeat segments. A single heartbeat consisted of 200 samples including the R-peak and the samples around the peak. This segment size contained maximum information of a single heartbeat and is shown in Figure 2.

### 2.3. Feature Extraction

In feature extraction, improved features based on DWT, RR interval, and Teager energy operator were selected, which were able to represent the morphological and dynamic changes in the ECG signal with more significance.

#### 2.3.1. Discrete Wavelet Transform (DWT)

Statistical features of biomedical signals usually change over position or time. Wavelet transform offers signal representation in both time and frequency domains, which makes it capable for analyzing quasiperiodic signals like ECG. Wavelet transform was employed in processing of the ECG signals for feature extraction [1], denoising [11], and heartbeat recognition [20]. In the proposed method, DWT was used as a feature extraction technique. After applying DWT, the ECG signal decomposed into low-frequency approximation components and high-frequency detail components.

The most commonly used wavelets which provide orthogonality properties are Daubechies, Coiflets, Symlets, and Discrete Meyer [21]. Each heartbeat was disintegrated using the finite impulse response (FIR) approximation of the Discrete Mayers wavelet transform. The frequency range of fourth-level approximation subband was 011.25 Hz, and the frequency range for fourth level detail subband was 11.2522.5 Hz. A total of 200 coefficients were extracted as wavelet features, which were processed using ICA for dimensionality reduction. Six major ICA components were selected from each of the two DWT subbands, resulting in a total of twelve morphological features from the two subbands.

#### 2.3.2. RR Interval Features

R is a point corresponding to the highest peak of the ECG waveform, and RR interval is the time between the successive QRS complexes. The ECG signal has a nonlinear dynamic behavior, and during arrhythmia, nonlinear dynamic components change more significantly than the linear counterparts. RR interval is simple, easy to calculate, and less prone to noise. Four types of RR interval features, namely, previous-RR, post-RR, average-RR, and local-RR interval, were derived from the RR sequence, to characterize the dynamic features of the heartbeat. The calculation of these features uses the following equations:

(2) $$\begin{array}{c} {\text{RR}}_{\text{pre}}\left(i\right)=\text{R}\left(i\right)-\text{R}\left(i-1\right),\\ {\text{RR}}_{\text{post}}\left(i\right)=\text{R}\left(i+1\right)-\text{R}\left(i\right),\\ {\text{RR}}_{\text{local}}=\frac{1}{10}\overset{5}{\underset{i=-5}{\sum }}\text{RR}\left(i\right),\\ {\text{RR}}_{\text{ave}}=\frac{1}{N_{\text{RR}}}\overset{N_{\text{RR}}}{\underset{i=1}{\sum }}\text{RR}\left(i\right), \end{array}$$

where *i* shows the location of the current R-peak and RR_pre_, RR_post_, RR_local_, and RR_ave_ represent the previous, post, local, and average RR interval, respectively. R(*i*) is the current R-peak, R(*i* − 1) and R(*i*+1) represent the previous and post R-peaks, respectively, and *N*_RR_ shows the total number of RR intervals in an ECG segment.

#### 2.3.3. Teager Energy Operator (TEO)

An independent analysis of RR interval does not capture the nonlinear nature of RR interval inconsistency. TEO was utilized to represent the nonlinear behavior of the RR interval, which is a nonlinear operator for energy tracking [17]. It measures the instantaneous frequency, amplitude envelope, and the energy of the system that generated the signal. The energy required by a source to generate signals with different frequencies and same energy and amplitude would be different. More energy is needed to generate a high-frequency signal as compared with a low-frequency signal. TEO reflects the source energy; hence, any instability in the conduction path and impulse generation gets revealed in the Teager energy function. For a discrete time signal *x*[*n*], the Teager–Kaiser nonlinear energy (NE) and the average nonlinear energy (ANE) in the time domain are given as

(3) $$\begin{array}{c} \text{NE}\left\{x\left[n\right]\right\}=x^{2}\left[n\right]-x\left[n-1\right]x\left[n+1\right], \end{array}$$

(4) $$\begin{array}{c} \text{ANE}=\frac{1}{N}\sum \text{NE}\left\{x\left[n\right]\right\}, \end{array}$$

where *N* represents the total number of samples in ECG heartbeat.

### 2.4. Neural Network (NN) Classifier

An artificial neural network consists of interconnected neurons which send and receive messages between each other. These interconnections are assigned weights, which represent a network state and are updated during the learning process. A feedforward neural network with 10 hidden layers was used for the classification of arrhythmia in this study. The network was implemented on MATLAB R2013a. The number of neurons in each hidden layer was limited to 50, which allowed training this network on a core-i5 CPU-based system with a RAM of 8 GB. An activation function based on rectified linear unit (ReLU) was used for the hidden layers, and a sigmoid function was used at the output layer. Back propagation with stochastic gradient decay was used for updating the network weights. The learning rate was optimized to a value of 0.63, using grid search for accuracy and to avoid over fitting.

## 3. Experimental Results

The details of the dataset used and the experimental results are presented in the following subsections.

### 3.1. Dataset

The MIT-BIH arrhythmia database (MITDB) [22] and MIT-BIH supraventricular arrhythmia database (SVDB) [23] from physionet were used for evaluation of the performance of the proposed algorithm. The MITDB includes 48 ECG recordings of 47 subjects, whereas the SVDB contains 78 half-hour ECG recordings. The data in SVDB were recorded to increase the supraventricular arrhythmias examples in MITDB. The databases contain an annotation file with locations of the “QRS” complex and the type of the heartbeat for each record. These class annotations for heartbeats were exploited as reference annotations for evaluation purpose of the proposed model.

### 3.2. Evaluation Strategy

Two different types of evaluation strategies were considered, namely, class-oriented [24] and subject-oriented [9]. In class-oriented strategy, all signals from both databases were segmented down using already annotated QRS locations. The resulting segments were divided into 18 different types of beats, namely, normal beat (NOR “N”), atrial premature contraction (APC “A”), fusion of ventricular and normal beat (FVN “F”), left bundle branch block (LBBB “L”), unclassifiable beat (UN “Q”), premature ventricular contraction (PVC “V”), right bundle branch block beat (RBBB “R”), ventricular flutter wave (VF “!”), atrial escape beat (AE “e”), fusion of paced and normal beat (FPN “f”), nodal (junctional) premature beat (NP “J”), isolated QRS-like artifact (—), aberrated atrial premature beat (AP “a”), ventricular escape beat (VE “E”), nodal (junctional) escape beat (NE “j”), nonconducted P-wave (blocked APB “x”), paced beat (PACE “/”), and supraventricular premature beat (SP “S”).

In addition, subject-oriented strategy was also evaluated. All 126 records from both the datasets were divided into a similar training and testing ratio as for the class-oriented scheme, but performance was reported according to ANSI/AAMI EC57:1998 standard [25]. The original 18 classes of heartbeats were grouped into five bigger classes, namely, nonectopic beats (N), ventricular ectopic beat (V), supraventricular ectopic beat (S), unknown beat (Q), and fusion beat (F). The mapping from the MITDB and SVDB classes to the ANSI/AAMI heartbeat classes is presented in Table 1.

The ECG signal from MITDB and SVDB datasets are first denoised to remove baseline wander. The denoised signal was segmented into different heartbeats of same length (200 samples each) by using R-peak location information in the given annotations. In total, 35875 beats form both databases (13724 from MIT-BIH arrhythmia dataset and 22151 beats from MIT-BIH supraventricular arrhythmia dataset) were considered. FIR approximation of Mayers wavelet was applied on each heartbeat segment. ICA was applied on the resulting 4^th^ level approximation and 4^th^ level detail coefficients independently to remove the redundancy between feature coefficients. In addition, dynamic features of the ECG signal were represented by four types of RR interval features, and Teager energy operator was used to represent the nonlinear dynamics of the ECG signal.

A 3-fold cross-validation method was used for training and testing of the classifier. The complete dataset (35,875 beats) was subsampled into two sets, one having 70% of the total samples and the other with the remaining heartbeats from each of the classes (50% data was selected for training from atrial escape beat (e), due to very low number of beats). 70% of total heartbeats were utilized for training purpose, and the rest of the heartbeats were used in testing and evaluation of the performance of the classifier. The process was repeated three times with different heartbeats used for training and testing. The average results of the three folds were calculated to assess the general performance of the proposed method. Sensitivity (Se), specificity (Sp), positive predictive value (PPV), and accuracy (Acc) were calculated to analyze the performance.

#### 3.2.1. Class-Oriented Evaluation

In class-oriented evaluation scheme, a neural network was trained to predict the class of test heartbeats among 18 different classes of arrhythmia. The specificity, sensitivity, and PPV of each individual class are summarized in Table 2, which shows that the proposed approach demonstrates a reasonable individual-class performance, showing greater specificity, good sensitivity, and PPV. Moreover, it has given better results on classes whose presence was low like “Q,” “e,” and “x.” The performance evaluation measures are shown for all three folds in Table 2, where the best accuracy achieved was 99.9%. In addition, 98.7% average sensitivity, 99.9% average specificity, 99.8% average PPV, and 99.75% overall accuracy were achieved. The confusion matrix is presented in Table 3, which shows the correctly classified and misclassified arrhythmia classes.

A comparison of the classification accuracy of the proposed approach and state-of-the-art methods based on class-oriented strategy is presented in Table 4. An improvement in accuracy with increased number of classes and reduced feature dimension is observed. In addition, other performance parameters such as sensitivity, specificity, and PPV have also shown improvement. These results depict that the proposed method is more generalized and computationally efficient for arrhythmia classification. An assessment based solely on “class-oriented” scheme is not a faithful measure for the performance analysis of a real-time heartbeat classification system. The performance of the subject-oriented scheme is also analyzed for practical evaluation.

#### 3.2.2. Subject-Oriented Evaluation

In subject-oriented scheme, results have reported according to the ANSI/AAMI standard. The specificity, sensitivity, PPV and accuracy of each individual class in ANSI/AAMI standard is shown in Table 5. The hybrid feature approach demonstrated reasonable individual-class performances, showing greater specificity, good sensitivity, positive PPV, and accuracy for all classes. The fusion beats showed lower PPV in Fold II, which can be attributed to smaller number of beats in this class. The peak accuracy achieved was 99.9% with 99.7% average sensitivity, 99.9% average specificity, 99.1% of average PPV, and 99.8% average accuracy.  Table 6 shows the confusion matrix depicting an improved performance on all classes with an average accuracy of 99.8%. A comparative analysis is presented in Table 7 with state-of-the-art techniques for subject-oriented classification. A significant improvement is observed, when taking this fact into account that 18 arrhythmia classes were classified. The comparison is reported for methods using the MIT-BIH data for adding credence to the results.

## 4. Conclusion

In this paper, a new technique for automatic heartbeat classification of all types of arrhythmia was presented. An improved hybrid feature representation of heartbeat segments was used based on a mixture of a set of derived morphological and dynamic features. The classification was done using twelve ICA projection coefficients computed from the DWT features, plus four RR interval features, and Teager energy value. Two types of evaluation schemes, class- and subject-oriented, were implemented for analyzing the system. On the standard benchmark of MIT-BIH arrhythmia database and MIT-BIH supraventricular arrhythmia database, an average accuracy of 99.75% with a peak accuracy in a single fold of 99.9% in the class-oriented evaluation was achieved. An accuracy of 99.8% in the subject-oriented evaluation was achieved. In future, an automatic patient customization scheme will be considered, allowing the heartbeat classification method to be able to adjust to individual physiological features using wearable sensors.

## Figures and Tables

**Table 1:** Mapping from MIT-BIH arrhythmia database (MITDB)/supraventricular arrhythmia database (SVDB) heartbeat classes to ANSI/AAMI heartbeat classes.

AAMI classes	MITDB/SVDB classes	Total
Nonectopic beat (N)	NOR, LBBB, RBBB, AE, NE	30929
Supraventricular ectopic beat (S)	APC, AP, APB, NP, SP	1538
Ventricular ectopic beat (V)	PVC, VE, VF	2035
Fusion beat (F)	F	14
Unknown beat (Q)	UN, FPN, PACE, ∣	1329

**Table 2:** A summary of performance analysis of the proposed method on each arrhythmia class in the “class-oriented” scheme.

	Fold I				Fold II				Fold III				Average			
Heartbeat type	Sp	Se	PPV	Acc	Sp	Se	PPV	Acc	Sp	Se	PPV	Acc	Sp	Se	PPV	Acc
Normal beat (NOR)	100	100	99.8	99.4	100	100	99.9	99.7	99.8	100	99.7	99.7	99.9	100	99.8	99.6
Atrial premature contraction	100	97	100	100	100	96.5	100	100	100	97.5	100	100	100	97	100	100
Fusion of ventricular and normal beat	100	100	100	100	100	100	100	92.7	100	100	100	100	100	100	100	97.5
Left bundle branch block (LBBB)	100	97.5	100	100	100	97	100	99.3	100	96.5	100	100	100	97	100	99.7
Unclassifiable beat (UN)	100	100	100	100	100	100	100	100	100	100	100	100	100	100	100	100
Right bundle branch block beat (RBBB)	99.8	99.4	99.4	99.7	99.9	99.2	99.4	99.4	99.9	99.3	99.4	99.4	99.8	99.3	99.4	99.5
Premature ventricular contraction (PVC)	100	99.1	99.6	99.7	100	99.2	99.6	99.5	100	99.3	99.6	99.6	100	99.2	99.6	99.6
Ventricular flutter wave (VF)	100	100	100	100	100	100	100	100	100	100	100	100	100	100	100	100
Aberrated atrial premature beat (AP)	100	100	100	100	100	100	100	100	100	100	100	100	100	100	100	100
Nodal (junctional) premature beat (NP)	100	92.6	100	100	100	92.8	100	100	100	92.7	100	100	100	92.8	100	100
Atrial escape beat (AE)	100	100	100	100	100	100	100	100	100	100	100	100	100	100	100	100
Fusion of paced and normal beat (FPN)	100	100	100	100	99.9	100	100	100	100	100	100	100	100	100	100	100
Isolated QRS-like artifact (Iso)	99.9	100	97.2	100	99.9	100	98.1	100	99.9	99.1	99.1	99.1	99.9	98.1	98.1	99.5
Ventricular escape beat (VE)	100	100	100	100	99.9	100	95.2	100	99.9	100	98.5	100	100	95.2	100	100
Nodal (junctional) escape beat	99.9	100	95	100	99.9	100	97.5	100	100	100	100	100	100	100	100	100
Paced beat (PACE)	99.9	100	99.2	100	100	100	100	100	99.9	100	99.6	100	100	99.2	100	100
Nonconducted P-wave (blocked APB)	100	100	100	100	100	100	100	100	100	100	100	100	100	100	100	100
Supraventricular premature beat (SP)	100	100	100	100	100	100	100	100	100	100	100	100	100	99.4	100	100
Average	99.9	99.9	99.4	99.9	99.9	99.3	99.4	99.3	99.9	99.9	99.8	99.9	99.9	98.7	99.8	99.75

**Table 3:** Confusion matrix for the proposed method using a neural network based classifier (class-oriented scheme).

Predicted labels	N	A	F	L	Q	R	V	!	a	J	E	f	—	E	J	/	X	S
*Actual labels*																		
N	8656	0	0	0	0	0	0	0	0	0	0	0	0	0	0	0	0	0
A	2	65	0	0	0	0	0	0	0	0	0	0	0	0	0	0	0	0
F	0	0	14	0	0	0	0	0	0	0	0	0	0	0	0	0	0	0
L	8	0	0	260	0	0	0	0	0	0	0	0	0	0	0	0	0	0
Q	0	0	0	0	5	0	0	0	0	0	0	0	0	0	0	0	0	0
R	2	0	0	0	0	314	0	0	0	0	0	0	0	0	0	0	0	0
V	4	0	0	0	0	0	564	0	0	0	0	0	0	0	0	0	0	0
!	0	0	0	0	0	0	0	21	0	0	0	0	0	0	0	0	0	0
a	0	0	0	0	0	0	0	0	1	0	0	0	0	0	0	0	0	0
J	1	0	0	0	0	0	0	0	0	13	0	0	0	0	0	0	0	0
e	0	0	0	0	0	0	0	0	0	0	1	0	0	0	0	0	0	0
f	0	0	0	0	0	0	0	0	0	0	0	33	0	0	0	0	0	0
—	0	0	0	0	0	2	0	0	0	0	0	0	105	0	0	0	0	0
E	1	0	0	0	0	0	0	0	0	0	0	0	0	20	0	0	0	0
j	0	0	0	0	0	0	0	0	0	0	0	0	0	0	40	0	0	0
/	0	0	0	0	0	0	0	0	0	0	0	0	2	0	0	254	0	0
x	0	0	0	0	0	0	0	0	0	0	0	0	0	0	0	0	2	0
S	0	0	0	0	0	0	2	0	0	0	0	0	0	0	0	0	0	376

**Table 4:** Comparison of the proposed scheme with state-of-the-art methods using class-oriented scheme.

	Features	Dimension	Classes	Accuracy	Sensitivity	Specificity	PPV
*Proposed*	*DWT + RR + TEO*	17	18	99.75	98.7	99.9	99.8
Zidelmal et al. [8]	Frequency content + RR + QRS	13	2	97.2	99	—	—
Ye et al. [11]	WT + ICA + RR	18	16	99.3	91.3	—	—
Ebrahimzadeh et al. [4]	HOS + timing interval	24	5	95.18	95.61	98.8	90.6
Pathoumvanh et al. [24]	DCT	5	5	99.11	97.01	99.44	—
Rabee and Barhumi [26]	Multi resolution WT	251	14	99.2	96.2	100	—
Alajlan et al. [27]	HOS of 2nd-order-cumulant	604	2	94.96	92.19	95.19	—
de Oliveira et al. [14]	Waveform + RR	—	2	95	95	99.87	98
Li et al. [19]	Timing interval + waveform amplitude	—	2	98.2	93.1	—	81.4

**Table 5:** Performance of the proposed method on each arrhythmia class in the “subject-oriented” scheme.

	Fold I				Fold II				Fold III				Average			
Heartbeat type	Sp	Se	PPV	Acc	Sp	Se	PPV	Acc	Sp	Se	PPV	Acc	Sp	Se	PPV	Acc
Nonectopic beats (N)	99.9	100	94.2	100	99.7	99.9	99.8	99.9	100	100	99.8	100	99.9	99.9	97.9	99.9
Supraventricular ectopic beats (S)	100	99.8	100	100	99.9	100	99.8	100	100	99.9	100	100	99.9	99.6	99.9	100
Ventricular ectopic beats (V)	100	99.6	100	100	100	99.5	100	99.9	100	99.7	100	100	100	99.6	100	99.9
Fusion beats (F)	99.9	100	99.9	100	99.9	100	92.8	98.9	99.9	100	100	100	99.9	100	97.6	99.6
Unclassifiable beats (Q)	100	99.6	100	99.8	100	99.4	100	100	100	99.5	100	99.7	100	99.5	100	99.8
Average	99.9	99.8	98.8	99.9	99.9	99.7	99.6	97.7	99.9	99.8	99.9	99.9	99.9	99.7	99.1	99.8

**Table 6:** Confusion matrix for Fold II using NN (subject-oriented scheme).

Predicted labels	N	S	V	F	Q
*Actual labels*					
N	9280	1	0	0	0
S	1	458	0	1	0
V	2	0	604	0	0
F	0	0	0	14	0
Q	2	0	0	0	398

**Table 7:** Comparison of the proposed scheme with state-of-the-art methods using subject-oriented scheme.

	Features	Dimensions	Classes	Accuracy	Sensitivity	Specificity
*Proposed*	*DWT + RR interval + TEO*	17	5 (18)	99.8	99.7	99.9
Ye et al. [11]	WT + ICA + RR	18	5 (16)	86.4	91.3	—
Martis et al. [9]	DWT + ICA	12	5 (15)	99.28	97.97	99.83
Mar et al. [15]	RR interval series and WT	—	3	93	80	82
de Lannoy et al. [5]	Waveform + HOS + RR	249	5 (16)	94	—	—

## Data Availability

The data used in this study are available for download at the physionet MIT-BIH website.
